# Ameliorative Effects of *Aquilaria malaccensis* Leaves Aqueous Extract on Reproductive Toxicity Induced by Cyclophosphamide in Male Rats

**DOI:** 10.21315/mjms2019.26.1.4

**Published:** 2019-02-28

**Authors:** Redzuan Nul Hakim Abdul Razak, Faridah Ismail, Muhammad Lokman Md Isa, Azantee Yazmie Abdul Wahab, Hussin Muhammad, Roszaman Ramli, Raja Arif Shah Raja Ismail

**Affiliations:** 1 Department of Basic Medical Sciences, Kulliyyah of Allied Health Sciences, International Islamic University of Malaysia, Jalan Sultan Ahmad Shah, Bandar Indera Mahkota, 25200 Kuantan, Pahang, Malaysia; 2 Department of Basic Medical Sciences, Kulliyyah of Medicine, International Islamic University of Malaysia, Jalan Sultan Ahmad Shah, Bandar Indera Mahkota, 25200 Kuantan, Pahang, Malaysia; 3 Department of Basic Medical Sciences, Kulliyyah of Nursing, International Islamic University of Malaysia, Jalan Sultan Ahmad Shah, Bandar Indera Mahkota, 25200 Kuantan, Pahang, Malaysia; 4 Department of Obstetrics and Gynaecology, International Islamic University of Malaysia, Jalan Sultan Ahmad Shah, Bandar Indera Mahkota, 25200 Kuantan, Pahang, Malaysia; 5 Herbal Medicine Research Centre, Institute for Medical Research, Jalan Pahang, 50588 Kuala Lumpur, Malaysia

**Keywords:** antioxidants, Aquilaria malaccensis, cyclophosphamide, oxidative stress, reproductive toxicity

## Abstract

**Background:**

Cyclophosphamide (CP) is a widely used anti-neoplastic and immunosuppressive agent that is associated with adverse side effects including reproductive toxicity. *Aquilaria malaccensis* (AM) is a traditional medicinal plant which was reported to exhibit high anti-oxidant and free radical scavenging properties. The present study was aimed to evaluate the protective effects of AM leaves extract on sperm quality following toxic exposure to CP.

**Methods:**

Forty-eight male Sprague Dawley rats were allocated into eight groups of six rats (*n* = 6): control, CP only (200 mg kg^−1^), AM only (100 mg kg^−1^, 300 mg kg^−1^ and 500 mg kg^−1^) and CP + AM (100 mg kg^−1^, 300 mg kg^−1^ and 500 mg kg^−1^). Animals were sacrificed after 63 days of treatment and the sperm from the caudal epididymis was taken for sperm analysis.

**Results:**

The body and the reproductive organs weight, sperm count and motility did not differ between CP and other groups (*P* > 0.05). A significant increase (*P* < 0.05) in percentage of the dead and abnormal sperm were seen in the CP alone treated group compared to the control group. Co-administration of AM to the CP exposed rats significantly reduced the (*P* < 0.05) percentage of abnormal sperm as compared to the CP only group.

**Conclusion:**

Overall, the present results represent the potential of AM to protect against CP induced reproductive toxicity.

## Introduction

Exposure to both therapeutic and toxic environmental agents during spermatogenesis may result in sperm disorders and infertility due to germ cell damage (1). Cyclophosphamide (CP) is a bifunctional alkylating agent used extensively for the treatment of various malignancies as well as an immunosuppressive agent for organ transplantation, systemic lupus erythematosus, multiple sclerosis and other benign diseases (2–5). CP has two active metabolites, phosphoramide mustard and acrolein. Phosphoramide mustard is responsible for the therapeutic activity, whereas acrolein adversely affects the human body by triggering toxic effects like cell death either apoptosis or necrosis and oncosis (6).

Despite the wide spectrum of clinical uses, CP treatment has been associated with many unfavourable complications including reproductive toxicity and infertility. Decline in sperm count (oligospermia and azoospermia) and an absence of spermatogenic cycles in testicular tissue have been demonstrated in adult patients receiving CP treatment (7). Previous studies on male rats also revealed the potential of CP to cause histological alterations in the testis and epididymis as well as disturbances in gonadotropin and testosterone secretion in the blood (8–10). In addition, chronic administration of CP at low doses in adult male rats was also reported to adversely affect the offspring by increasing pre- and post-implantation loss, inducing fetal malformations as well as growth retardation (11).

Although the exact mechanism by which CP causes testicular injury is still unknown, numerous studies have shown that the oxidative stress-induced biochemical and physiological disturbances are responsible for the CP toxicity in the testis and spermatozoa (12). The CP metabolite, acrolein has been proven to increase lipid peroxidation, induce oxidative damage, cytoskeletal abnormalities, and decrease the viability of Sertoli cells. Furthermore, sperm are particularly vulnerable to lipid peroxidation due to the abundance of polyunsaturated fatty acids (PUFA) in their membrane and a lack of antioxidant content in their cytoplasm (13). Various strategies have been proposed to preserve fertility following CP-induced damage. Many studies have suggested that CP-mediated testicular injury can be prevented by the administration of compounds with antioxidant properties (14, 15).

*Aquilaria* species (agarwood) from the *Thymelaeaceae* family is one of the most valuable plants on earth and has been used in religious, aromatic and medicinal preparations for thousands of years (16, 17). It is a large evergreen tree growing over 15 m–30 m in height and 1.5 m–2.5 m in diameter and is able to produce fragrant agarwood resins when the wood is infected by pathogens or wounded (18). In Malaysia, the most popular *Aquilaria* species consumed and for trade is *A. malaccensis* (AM). It is well distributed in our country and is the main genus to produce Agarwood or ‘gaharu’ (19). In folk medicine, this plant is commonly used in inflammatory-related ailments such as arthritis, asthma, gout, acting as an aphrodisiac and stimulant as well as a carminative agent (18). Recently, a variety of their parts including the leaves, skin, seeds, wood and roots were found to be beneficial in the pharmaceutical field. For example, the leaves of *Aquilaria sp.* were reported to exhibit potent anti-oxidant, anti-microbial, analgesic, antipyretic, anti-inflammatory and anti-hyperglycemic activities (20–24).

Although this plant had been reported to have medicinal value towards various kinds of diseases, information on its effect on the male fertility and reproduction is very scarce. Therefore, the present study was undertaken to evaluate the potentials of AM in improving sperm quality following toxic paternal exposure to cyclophosphamide.

## Materials and Methods

### Plant Material

The leaves were collected from an AM tree plantation in the Forest Research Institute Malaysia (FRIM) at Kepong, Selangor. The specimen voucher PIIUM 0296 was prepared and deposited at the Natural Medicinal Product Centre, Kulliyyah of Pharmacy, International Islamic University Malaysia (IIUM).

### Preparation of the Aqueous Extract

After collection, the fresh leaves were dried in a drying oven at 40 °C and powdered using an electric blender. Dried leaf powder weighted 150 g was soaked in 1500 mL of 40 °C hot distilled water and placed in sonicator for 30 min. The hot-water extract was filtered through a Whatman No. 1 filter paper and the extraction steps were repeated twice. The resulting filtrates were then freeze-dried and the powder yield was stored at 4 °C in an airtight bottle until further use. The average (W/W) yield was 10%.

### Animal

Forty-eight male Sprague Dawley rats aged 2–3 months old with weights between 150 g–200 g were selected and acclimatised for a week before starting the treatment. The animals were kept in polycarbonate cages inside a well-ventilated room at a temperature of 22 °C±2 °C, maintained under standard laboratory conditions with 50%±10% humidity and a cycle of 12 h light and 12 h dark. Standard laboratory animal feed and water were provided ad libitum. All ethical themes of studies on animals were considered carefully, and the experimental protocol was approved by the Institutional Animal Care and Use Committee of the International Islamic University Malaysia (IACUC-IIUM) with the reference number IIUM/IACUC-Approval/2017 (15).

### Experimental Protocol

The male rats were randomly divided into eight groups consisting of six animals each. The CP was administered to the rats by intraperitoneal injection in a single dose (200 mg kg^−1^ body weight), only once on day 1 of treatment. AM leaves extract was dissolved in distilled water and administered to the rats by oral gavage daily. Treatment group were as follows:

1. Control (distilled water, p.o.)
2. CP (200 mg kg^−1^, single dose, i.p.)
3. AM-100 (100 mg kg^−1^/day, p.o.)
4. AM-300 (300 mg kg^−1^/day, p.o.)
5. AM-500 (500 mg kg^−1^/day, p.o.)
6. CP+AM-100 (200 mg kg^−1^ CP, single dose, i.p. + 100 mg kg^−1^/day AM, p.o.)
7. CP+AM-300 (200 mg kg^−1^ CP, single dose, i.p. + 300 mg/kg/day AM, p.o.)
8. CP+AM-500 (200 mg kg^−1^ CP, single dose, i.p. + 500 mg kg^−1^/day AM, p.o.)

All groups were treated for 63 days. Each male rat was weighed on a precision balance daily. In addition, the food and water intake, the consistency of stool and the colour of urine were inspected daily.

### Animal Sampling

Male rats were euthanised on the 64th day of the treatment by injection of sodium pentobarbital (60 mg kg^−1^) intraperitoneally and their epididymis and testis were removed. The tissues were placed on sterile paper to blot away any blood and fluid and weighed on a precise balance. Then, the caudal part of the epididymis was removed and placed in a petri dish containing 4 mL of sperm washing media and finely minced with a scissor. The sperm suspension was incubated for 30 min on hot plate at 37 °C to allow the sperm to swim out before proceeding with sperm analysis

### Sperm Motility

One drop of epididymal sperm suspension was placed on a pre-warmed microscope slide at 37 °C and covered with a coverslip. At least 10 microscope fields were observed at 400x magnification using a phase contrast microscope (25). Assessment of the sperm near the edge of the coverslip was avoided. The percentage of motile sperm was recorded and sperm motility was expressed as a percentage of motile sperm of the total sperm counted (26).

### Sperm Count

Sperm count was carried out by using a haemocytometer. The counting was done in the ruled squares on the slide. Firstly, 10 μL was pipetted from the epididymal suspension and mixed with 90 μL distilled water to create a ten-fold dilution. Then, 10 μL from the sperm water mixture was placed into the counting chamber on the haemocytometer and covered with a cover slip. The number of spermatozoa with head and tail in five squares (four corners and the centre) in the centre grid of both sides were counted and averaged. Below is the formula used to obtain the sperm concentration (sperm count) in every mL (27).

$$\begin{array}{l} \text{Concentration}\;\text{of}\;\text{sperm}=\text{Number}\;\text{of}\\ \text{spermatozoa}\;\text{in}\;\text{five}\;\text{squares}\times 10\;(\text{dilution})\times \text{five}\\ \text{squares}\times 10000 \end{array}$$

#### Sperm Viability

An aliquot of 50 μL of sperm suspension was mixed thoroughly with 50 μL of eosin-nigrosin stain in a clean Eppendorf tube. Fifteen microlitre of the stained sperm mixture was transferred onto a glass slide and five smears were made for each rat. The glass slides were left to dry at room temperature. Coverslips were placed with one drop of mounting medium before being observed under x100 oil immersion with a bright field microscope. The dead sperm showed a pink coloration of the head whereas the viable sperm showed a whitish or colorless head. Approximately 200 sperms were observed for dead and live cells and the percentage of the dead and live cells were recorded (28).

### Sperm Morphology

Sperm morphology analysis used the same sperm smear made for the sperm viability analysis. The sperm were observed under x100 oil immersion magnification of imaging microscope to clearly evaluate the morphology of the sperm head, neck and tail. Two hundred sperm were examined in order to classify them into a normal or abnormal type of sperm. The abnormalities were also categorised into head or neck abnormalities (28).

### Statistical Analysis

We justified our sample size. The sample size was calculated based on a previous study using a formula from www.openepi.com for comparing two means. Based on an assumption that the mean difference between the sperm parameters of the CP group and CP + AM is 10, standard deviation at 5, the level of significance at 5%, power of study at 80% and attrition rate of 30%, the minimum number of rats required is six for each group (25). Results were expressed as mean (SD) and median (interquartile range) as indicated. Statistical evaluations were performed using One-way ANOVA parametric test followed by Tukey test as the post-hoc test for the normally distributed data and Kruskal-Wallis non-parametric test followed by Dunn’s pairwise test as the post hoc test for the abnormally distributed data. SPSS 21.0 software was used for statistical analysis and *P*-value < 0.05 was considered as significant.

## Results

### Clinical Signs, Body and Organ Weight

CP treated animals had shown general signs of deterioration such as hair loss, lethargy, hunched posture, low activity and hematuria. The weights of body, testes and epididymis were measured on the day after full treatment. Data for the total body weight, absolute and relative weight of both the testis and epididymis were normally distributed and the results are presented in Table 1. There was a statistically significant difference in mean body weight between the groups as determined by one-way ANOVA [*F*(7,34) = 5.359, *P* < 0.001]. A Tukey post hoc test revealed that AM administration alone at dose 500 mg kg^−1^ and administration of AM at all doses to the CP-exposed group had significantly reduced (*P* = 0.004, *P* = 0.012, *P* = 0.011 and *P* = 0.004, respectively) the body weight when compared to the control group. However no significant differences were seen in the mean body weight of the CP treated group compared to control or other groups. For the reproductive organs weight, no significant difference was seen in the absolute weight of the testis and the relative weight of the testis and epididymis between the groups [*F*(7,34) = 1.358, *P*= 0.255, *F*(7,34) = 0.603, *P* = 0.749 and *F*(7,34) = 0.429, *P* = 0.877, respectively]. For the absolute epididymis weight, there was a significant difference between the groups [*F*(7,34) = 2.788, *P* = 0.021], and the post hoc test indicated that the CP+AM-500 group had a significantly lower (*P* = 0.047) absolute epididymis weight compared to the AM-100 group. However, there was no significant effect observed between the groups in comparison to the control and CP group.

### Sperm Count

Table 2 presents the results for the sperm analysis including sperm count, motility, viability and morphology. Data for the sperm count was not normally distributed and the Kruskall-Wallis test indicated that there was no significant difference (*P* = 0.543) in the mean sperm count between the groups. However, descriptively, the CP only group seemed to possess the lowest number of sperm count compared to control and other groups. While co-administration of AM to the CP exposed groups helped to slightly increase the sperm count especially at the dose of 500 mg kg^−1^/day. The results also showed that the groups receiving AM only at all doses had a lower sperm count compared to the control group.

### Sperm Motility

Data for the sperm motility was normally distributed. As shown in Table 2, there was no significant difference in sperm motility between the groups as determined by one-way ANOVA test [*F*(7,32) = 1.987*, P* = 0.088]. However, descriptively, it was indicated that the CP only group had the lowest sperm motility compared to those of the control, AM and CP+AM groups. Supplementation of the CP exposed group with AM slightly increased the sperm motility with the highest improvement seen with AM given at dose 500 mg kg^−1^. In addition, sperm motility was also found to be lower in the AM group at all doses compared to the control group.

### Sperm Viability

Data for sperm viability was not normally distributed and a Kruskal-Wallis test was applied to assess for significant differences. Table 2 showed that there was strong evidence of a difference (*P* = 0.001) in the mean sperm viability between the groups. Dunn’s pairwise test was carried out which showed statistically higher sperm viability in the control (*P* = 0.05, adjusted using the Bonferroni correction) and AM-100 (*P* = 0.05, adjusted using the Bonferroni correction) groups when compared to the CP group. Additionally, although not statistically significant, co-treatment of CP with AM at all doses seemed to improve the sperm viability when compared to the CP only group.

#### Number of orphologically abnormal sperm

**Figure 1:**
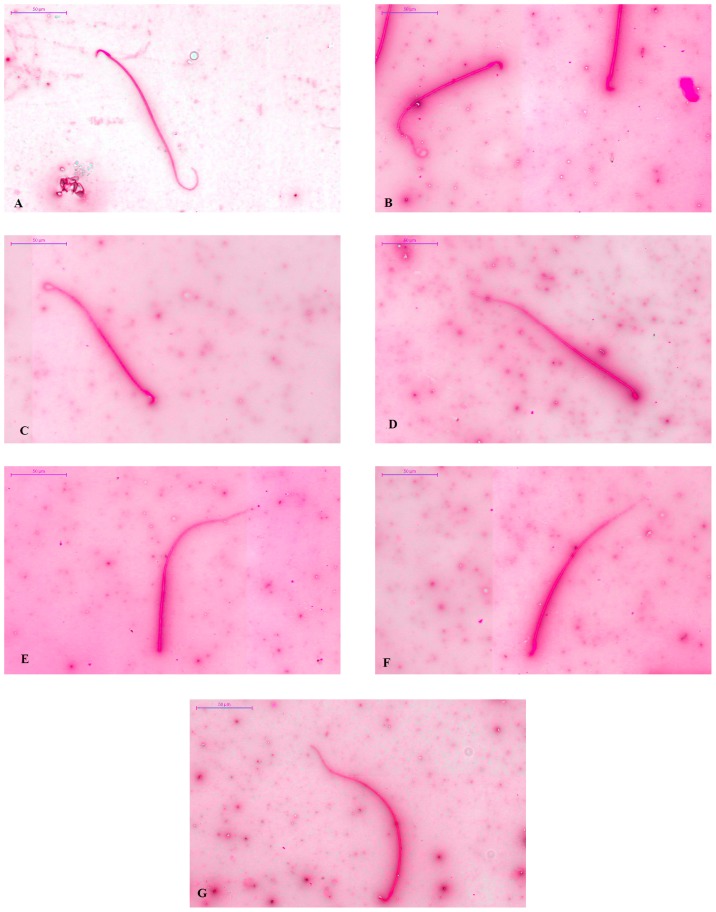
(A) normal sperm head and tail of control group, (B) sperm with coiled tail of cyclophosphamide (CP) alone group, (C) sperm with folded tail of CP alone group, (D) sperm with amorphous head of CP alone group, (E) headless sperm of CP alone group, (F) banana-shaped head of CP alone group, (G) normal sperm head and tail of the CP treated groups supplemented with AM leaves extract 100 mg kg^−1^/day

Data for the number of abnormal sperm were normally distributed and as shown in Table 2, statistically significant difference was observed between the groups when assessed using one-way ANOVA [*F*(7,34) = 5.356, *P* < 0.001]. Tukey post hoc test revealed that the treatment of male rats with CP only and AM only at dose 500 mg kg^−1^ caused a significant increase (*P* < 0.001 and *P* = 0.038, respectively) in the number of abnormal sperm compared to those of the control. Co-administration of AM after exposure to CP at dose 100 mg kg^−1^/day caused significant reduction (*P* = 0.018) in the number of abnormal sperms as compared to the CP only group. Apart from that, groups receiving AM at doses 100 mg kg^−1^/day and 300 mg kg^−1^/day also had significantly lower (*P* = 0.009 and *P* = 0.023, respectively) abnormal sperm number compared to the CP treated group. As shown in Table 3, two types of abnormalities were evaluated in this study, the head and tail abnormalities. For the number of abnormal heads, data was abnormally distributed and the Kruskall-Wallis test indicated no significant difference between the groups (*P* = 0.169). For the number of abnormal tails, data was normally distributed and the one-way ANOVA test showed a presence of significant difference between the groups [*F*(7,34) = 5.178, *P* < 0.001]. From the results, it can be concluded that most abnormalities were seen in the sperm tail and the CP group had a significantly higher (*P* < 0.001) number of abnormal sperm tail compared to the control group. While supplementation of AM at dose 100 mg/kg/day after CP exposure (CP+AM-100) significantly reduced (*P* = 0.029) the number of abnormal sperm tail. Figure 1 shows a representation of the normal and abnormal sperm head and tail of the rats in various treatment groups.

## Discussion

The biochemical basis of CP toxicity is believed to be associated with free radical generation in the affected tissues. Many studies have demonstrated that CP treatment causes induction of oxidative stress by the generation of free radicals and reactive oxygen species (ROS) (12, 29, 30). Excessive amounts of ROS production trigger DNA fragmentation and defective sperm function associated with peroxidative damage to the mitochondria and sperm membrane. Moreover, spermatozoa are particularly vulnerable to ROS attack because their cell membranes are rich in PUFA, which can be oxidised (lipid peroxidation), and their cytoplasm possesses only a small concentration of antioxidant enzymes to help neutralize ROS (31, 32).

Previous studies have demonstrated that CP treatment in male rodents resulted in significant decrease in body weight, testis weight and epididymis weight (8, 9, 33). Testicular weight depends mainly on the mass of spermatogenic cells and its reduction indicates severe decrease in sperm production and Leydig cells atrophy (8). However, in the present study, no changes in body weight or reproductive organs weight were seen in the CP treated group in comparison to the control group. These findings are in agreement with the results of Satoh et al. (34) and Fukushima et al. (35). The inconsistent findings of the effects of CP on body and reproductive organs weight in the previous studies are possibly due to the differences in the dose used, the duration of treatment, the route of CP administration and the sensitivity of the animals used (36). Additionally, our findings also showed that AM administration at high dose (500 mg kg^−1^/day) and AM co-administration at all doses to CP group significantly reduced the rat’s body weight. A study has demonstrated that the administration of *Aquilaria crassna* leaves extract at a high dose for seven days significantly reduced the body weight in rats. This finding may be explained by the presence of a laxative effect and α-glucosidase inhibitor activity in agarwood leaves extract which can interfere with the carbohydrate absorption in the body (22, 37–39).

CP treatment is also known to cause long-term or permanent azoospermia, which is most likely the result of spermatogonial stem cell death (40, 41). These rapidly dividing differentiating spermatogonia are found to be more susceptible to damage by cytotoxic chemotherapeutic drugs than the later stage germ cells. Thus, toxic exposure of these stem cells to CP can lead to the failure of spermatogenesis and loss of sperm production (41). While the duration of azoospermia appears to be influenced by the proportion of spermatogonia killed. If all of these cells are killed, the azoospermia will be irreversible and vice versa (8). CP can also compromise the spermatogenesis process by affecting the pituitary luteinizing hormone regulation, leading to inhibition of Leydig cell testosterone production and consequently, low testosterone levels in the blood (42). In our study, although CP alone group seemed to possess the lowest number of sperm count in the epididymis, no significant difference was observed between the groups. This may indicate a sign of recovery from the lesions induced by CP. Two processes are involved in recovery from the damaged germinal cells in the testis, one is regeneration, the increase in number of spermatogonial stem cells; and the other is repopulation, the differentiation of spermatogonia and the refilling of tubules by their progeny (43). Thus, our finding is in parallel with earlier reports that sperm production can partially recover from gonadotoxic effects after a gap of several weeks (44, 45). It can also be concluded that the existence of surviving spermatogonial stem cells following cytotoxic damage is essential for recovery of normal spermatogenesis (44). From our study, a small increment in the concentration of sperm was observed in the CP group supplemented with AM especially at doses of 500 mg kg^−1^ compared to the CP only group. As previously explained, the damage of the male germ cells by CP occurs due to the oxidative damage to PUFA of the cells membrane, leading to disruption of its permeability and increased apoptosis at specific stages of the germinal cycle (9). Thus, the improvement in sperm count in this group indicates that AM may be beneficial to protect spermatogonial stem cells against oxidative damage induced by CP and subsequently improve the concentration of sperm produced.

Another important parameter of male fertility are sperm motility and viability. CP exposure was reported to cause impaired sperm motility and viability presumably due to the disturbance of sperm flagellum function, the important machinery for motility of sperm cells through rapid loss of intracellular adenosine triphosphate (ATP) (42, 46). Alteration of the testicular tricarboxyclic acid cycle enzyme activities was also demonstrated in the CP-treated rats, which can lead to impaired energy metabolism (47). ATP is the common form of energy used for cellular metabolism and is necessary for sperm motility and fertilization. It is synthesized either by glycolysis in the cytoplasm or through oxidative phosphorylation in the mitochondria (48). ATP that is generated by oxidative phosphorylation in the mitochondrial membrane will be transferred to the microtubules to drive motility in sperm (49). Hence, oxidative damage to mitochondrial DNA (mtDNA) in sperm can lead to impaired sperm motility. Disturbance in membrane pump function and cellular Ca^2+^ homeostasis resulting from the loss of sperm membrane fluidity after ROS attacks is another occurance that can affect the sperm motility and viability (50). Apart from that, CP can exert direct toxicity to the spermatogenic compartment and alter the testicular antioxidant enzyme activity. As mentioned before, antioxidant enzymes play a crucial role in normal differentiation and development of spermatogonial cells to mature spermatozoa by protecting the cells from ROS injury (9, 42). Thus, suppression of these antioxidant enzymes activities can lead to defective sperm structures and generation of non-viable sperm. In present study, although there was no statistically significant difference in terms of sperm motility between the test groups, the CP treated group still showed the least percentage of motile sperm. The number of viable sperm was also significantly reduced in the CP treated group compared to the control group. This finding is in line with previous studies that indicate CP decreases sperm motility and viability in rats (46, 51). Additionally, an increment in number of motile and live sperm was seen in CP groups supplemented with AM especially at doses 300 mg kg^−1^/day and 500 mg kg^−1^/day compared to the CP only treated group. This discovered improvement indicates that the high antioxidant activity of AM may possess curative effects by scavenging the dangerous ROS induced by CP and prevent the mitochondrial damage.

Abnormalities in sperm morphology are also commonly observed following CP treatment. A study conducted by Ilbey et al demonstrated that CP administration to rats at a single dose of 100 mg kg^−1^ body weight significantly increased the number of abnormal and dead sperms (25). While Tripathi and Jena (33) revealed that CP treatment had given rise to abnormalities in the sperm head morphology in a dose-dependent manner. Our finding is in agreement with the above reports, whereas significant increases in total and tail abnormality of sperm were observed in the group receiving CP treatment alone when compared to the control group. The increased morphological defects in sperm is possibly due to the direct effect of CP on the cellular DNA, which is the primary target of CP for its anti-neoplastic and toxic activity (29). CP metabolites, phosphoramide mustard can bind to the N-7 position of guanine and to the phosphate backbone of DNA, leading to induction of a mixture of interstrand, DNA protein cross-links and cells enlargement (8). Lesions in germ cell DNA increased severely throughout the maturation in the reproductive tract and impaired the differentiation of spermatozoa (52). In addition, the toxic effect of CP on sperm morphology could also be a result from peroxidation of PUFA in the plasma membranes of spermatozoa by free radicals (29). Aprioku (50) had reported that peroxidation of critical thiol groups in protein can alter the structure and function of spermatozoa. Our study also demonstrated that the administration of AM at dose 100 mg kg^−1^ following treatment with CP resulted in a statistically significant decrease in the presence of total and tail sperm abnormalities. The rationale for the prevention of CP-induced sperm abnormalities by AM is in its ability to scavenge free radicals that cause lipid peroxidation and oxidative DNA damage.

It is well known that antioxidants form an important part in a cells defense against free radical damage. Several reports have revealed the benefit of antioxidants in protecting the male reproductive system from the adverse effects of ROS produced during CP exposure. For instance, it was found that ascorbic acid and several herbs with strong antioxidant properties such as *Crataegus monogyna* and Yukmijihwang-tang can improve reproductive toxicity of CP through reduction of oxidative stress (9, 53, 54). The present study showed that AM leaves extract has the potential to ameliorate the toxicity induced by CP and improve the sperm parameters. As far as we are concerned, this is the first study to assess the effects of AM leaves extract on sperm quality. A study had been done before to evaluate the effect of steroid isolated from the bark of AM on male rats’ reproduction and it revealed an improvement in sexual activity, sperm count and motility following the steroid administration (55). The possible mechanism of AM protection against testicular injury is attributed to its high antioxidant effect. Studies on different extracts of AM leaves including hexane, methanol, ethyl-acetate, and water using 2, 2-diphenil-1-picrylhydrazyl (DPPH) showed that the extracts exhibited strong antioxidant and free radical scavenging activity (20, 21, 56, 57). In addition, increasing evidences support the fact that AM is advantageous where free radicals are known to play a predominant role in toxicity including cancer and diabetes. Previous study revealed that AM posess cytotoxic activity toward cancer cell lines and can serve as an alternative treatment for human colon cancer (58). In another study, anticancer activity towards MCF-7 breast cancer cells and HeLa cervical cancer cells were observed (59, 60). AM leaves extract also helps to improve blood glucose levels in diabetic rats and efficiently inhibit α-glucosidase and α-amylase enzymes in vitro. All these important pharmacological properties of agarwood is attributed to the presence of many bioactive chemical constituents such as alkaloids, tannins, phenols, terpenoids, quinones and flavonoids (38). These phytochemicals which are found to be highest in leaf extracts have high antioxidant activity where they provide protection against damage and substantially reduce the risk of developing chronic disease (20).

Overall, our results indicated that significant improvement in sperm parameters of CP-treated rats was mostly observed with AM supplementation at dose 100 mg kg^−1^/day. Although other AM doses (300 mg kg^−1^/day and 500 mg kg^−1^/day) improved the sperm parameters, the results were statistically insignificant. In addition, administration of AM alone at all doses resulted in lower sperm quality compared to the control group. Generally, AM leaves aqueous extract administration is considered safe and the AM dosage should not cause any negative effects on sperm. Previous study indicated that AM leaves extract administration up to 520 mg kg^−1^ daily for 90 days in male mice does not cause any toxic signs and symptoms and is safe for consumption (61). The LD50 of the leaf extract was also found to be above 2000 mg kg^−1^ (62). Therefore more studies are needed to elucidate the protective mechanism of AM on sperm, to identify active compounds responsible for the effects and to assess the interaction of all phytochemicals present in AM and their effects on sperm. Replication of the study with higher sample size is also suggested to prove the validity of the findings

## Conclusion

In conclusion, the findings of our study indicate that CP induces significant oxidative stress to the testis and impairs the sperm quality. AM supplementation following paternal CP exposure has the potential to protect the male gametes from cellular and structural damage by scavenging free radicals, inhibiting the oxidative processes or by enhancing the antioxidant enzyme activity in male gonads. Therefore, AM may be a potential therapeutic agent against CP induced reproductive toxicity.

## Figures and Tables

**Table 1:** Effect of cyclophosphamide and *Aquilaria malaccensis* leaves aqueous extract on body and weights of testis and epididymis

	Final Body Weight (BW, g)	Absolute organ weight (g)		Relative organ weight (per BW, %)	
	
		Testis	Epididymides	Testis	Epididymides
Control	440.65 (18.89)	3.51 (0.280)	1.26 (0.160)	0.80 (0.073)	0.29 (0.044)
CP	397.11 (24.99)	3.35 (0.327)	1.23 (0.080)	0.85 (0.086)	0.31 (0.034)
AM-100	422.39 (34.63)	3.50 (0.231)	1.29 (0.177)	0.83 (0.080)	0.31 (0.046)
AM-300	406.76 (35.73)	3.40 (0.120)	1.25 (0.116)	0.84 (0.070)	0.31 (0.025)
AM-500	354.33 (17.17)*	3.17 (0.365)	1.12 (0.145)	0.89 (0.105)	0.32 (0.044)
CP+AM-100	366.90 (35.43)*	2.99 (0.831)	1.13 (0.198)	0.80 (0.171)	0.31 (0.030)
CP+AM-300	366.79 (47.81)*	3.26 (0.422)	1.08 (0.049)	0.90 (0.191)	0.30 (0.049)
CP+AM-500	359.77 (27.66)*	3.07 (0.123)	1.02 (0.058)	0.86 (0.041)	0.29 (0.034)
*F*-statistic	5.359	1.358	2.788	0.603	0.429
*P*-value^a^	0.000	0.255	0.021	0.749	0.877

The values are expressed as mean (SD),

a One way ANOVA with post-hoc Tukey test

* Significant differences as compared with the control group at *P* < 0.05

† Significant differences as compared with the CP group at *P* < 0.05

Abbreviation: CP = Cyclophosphamide; AM-100 (*Aquilaria malaccensis* 100 mg kg^−1^/day), AM-300 (*Aquilaria malaccensis* 300 mg kg^−1^/day), AM-500 (*Aquilaria malaccensis* 500 mg kg^−1^/day)

**Table 2:** Effect of cyclophosphamide and *Aquilaria malaccensis* leaves aqueous extract on epididymal sperm characteristics

	Sperm count (10^7^/mL)	Sperm motility (%)	Sperm viability (%)	No. of abnormal sperm (%)
Control	8.37 (7.51)	68.06.(11.46)	100 (0.00)†	5.67 (3.61)†
CP	2.09 (1.87)	41.25 (14.17)	65.40 (33.38)*	28.20 (11.30)*
AM-100	2.36 (2.23)	55.45 (17.45)	84.17 (38.78)	12.83 (6.79)†
AM-300	4.93 (5.08)	64.70 (11.82)	100 (0.00)†	14.17 (6.24)†
AM-500	2.48 (0.77)	49.29 (12.95)	98.75 (2.50)	19.75 (7.80)*
CP+AM-100	2.60 (1.98)	42.32 (21.44)	80.00 (39.52)	13.20 (4.15)†
CP+AM-300	2.79 (2.97)	46.57 (12.49)	97.20 (1.68)	18.00 (4.47)
CP+AM-500	3.39 (3.02)	50.83 (21.34)	93.70 (4.86)	18.00 (5.39)
*F***-**statistic	–	1.987	–	5.356
*P***-**value	0.543^b^	0.088^a^	0.001^b^	0.000^a^

The values are expressed as mean (SD),

a One way ANOVA with post-hoc Tukey test,

b Kruskal-Wallis test with post hoc Dunn’s pairwise test

* Significant differences as compared with the control group at *P*<0.05

† Significant differences as compared with the CP group at *P*<0.05

Abbreviation: CP = Cyclophosphamide; AM-100 (*Aquilaria malaccensis* 100 mg kg^−1^/day), AM-300 (*Aquilaria malaccensis* 300 mg kg^−1^/day), AM-500 (*Aquilaria malaccensis* 500 mg kg^−1^/day)

**Table 3:** Effect of cyclophosphamide and *Aquilaria malaccensis* leaves aqueous extract on sperm head and tail abnormalities

	No. of abnormal head	No. of abnormal tail
Control	0.67 (0.82)	5.00 (3.41)†
CP	2.20 (1.92)	26.00 (10.89)*
AM-100	1.33 (1.51)	11.50 (5.86)†
AM-300	2.83 (2.93)	11.33 (6.68)†
AM-500	0.50 (0.58)	19.25 (7.81)*
CP+AM-100	1.00 (1.00)	12.20 (3.90)†
CP+AM-300	1.80 (1.10)	16.20 (3.63)
CP+AM-500	2.60 (1.14)	15.40 (5.77)
F-statistic	-	5.178
P-value	0.169^b^	0.000^a^

The values are expressed as mean (SD),

a One way ANOVA with post-hoc Tukey test,

b Kruskal-Wallis test with post hoc Dunn’s pairwise test

* Significant differences as compared with the control group at *P* < 0.05

† Significant differences as compared with the CP group at *P* < 0.05

Abbreviation: CP = Cyclophosphamide; AM-100 (*Aquilaria malaccensis* 100 mg kg^−1^/day), AM-300 (*Aquilaria malaccensis* 300 mg kg^−1^/day), AM-500 (*Aquilaria malaccensis* 500 mg kg^−1^/day)
